# Chicago Medical Society, Meeting of January 18th

**Published:** 1886-03

**Authors:** 


### CHICAGO MEDICAL SOCIETY.

*Stated Meeting, January 18th, 1886.*—THE PRESIDENT, PRO TEM., D. W. GRAHAM, M. D., in the chair.

DR. E. J. DOERING read a paper entitled "MUTUAL PROTECTION AGAINST BLACKMAIL." The author stated that among the many trials which physicians have to encounter in the practice of their profession is the ever-existing liability of being blackmailed. This may either assume the more frequent form of a so-called malpractice suit, or the relatively less frequent charge of a criminal assault, according to the viciousness of the complainant. Such suits against physicians are increasing. One reason quoted was the fact that every city is overrun with petty lawyers, who have little or nothing to do, and are always willing to encourage any suit whatever, if there be the least prospect of getting something out of the defendant. The author stated that since investigating the matter he became convinced that many of these blackmail schemes were settled before being made public. Many a physician preferred being robbed of one or two hundred dollars, rather than incur the publicity, the loss of time, and the endless expense of a lawsuit. Again, the average jury, composed of the ignorant and illiterate, will always have a strong leaning toward the complainant, and against the defendant, in a malpractice suit, as physicians are popularly supposed to be capitalists. The author stated that personally he had never been sued or even threatened with a suit, and it was therefore from no motive of selfish interest, but from a sincere regard for the welfare of the profession, that he advocated the formation of an association for the mutual protection of physicians against blackmailing suits of all kinds. His plan is to organize a society composed of two or three hundred members of the regular profession, all of whom shall be of acknowledged ability, possessing a good moral character and standing in the community. Said association to employ the best legal talent attainable, by the year, to furnish the members such legal advice as they may desire at any time, and defend any suit against the members arising in the discharge of their professional duties. It was stated that the expense to each member of an association composed of about two hundred would not exceed five dollars per annum, and that the initiation fee of five dollars would create a sufficient fund for court expenses. Such an association would be a power in preventing suits. Let it be known that the individual physician is backed by the financial and moral support of a few hundred of the best physicians, and aided by the best legal talent obtainable, and he will be let alone by the offscourings and dregs of society who constitute, almost without exception, the blackmailing element in our professional life. The author stated that he was not aware of the existence of such an association as the one proposed, in any city, but the principle, at least, had been carried out recently by the New York Medical Society, in voting $500 to assist in the defense of the Drs. Purdy, members of the society, in the case of Brown *vs.* Purdy. After reading a number of letters from prominent physicians in favor of forming a protective association, and presenting several legal opinions sustaining the advisability, practicability and legal status of such a society, the author concluded by stating his firm belief that such an association for mutual protection is needed; that it would be a power for good; that it would draw the profession closer together; that, in short, it would be based on the principles of a common brotherhood, viz.: equality, harmony, justice and unity.

DR. F. C. HOTZ said that the extract of his letter to Dr. Doering, which was incorporated in the paper, indicated that at the time it was written he did not think favorably of the project. And, after listening with much interest to the doctor's arguments, he saw no reason for changing his opinion. Professional reputation and honor is the most personal of all personal property; if he lost it, it does not hurt anybody but himself, and therefore if any attack be made on it he should certainly wish to employ among the able lawyers the one in whose ability he had the greatest confidence. But he was not sure whether the lawyer retained by this protective union would be the one to whom he should like to trust the defense of his reputation. The attorney might be as able, or abler, than the lawyer of his own choice; but should the case go against him, he should never feel satisfied that the lawyer had done all that could be done for him unless he had full confidence in him. It is with the lawyer as with the physician, a question of confidence, and his patrons find no fault with his treatment as long as they have implicit faith in his ability.

An objection of greater weight, however, has been urged by several of the doctor's correspondents in asking what possible effect it might have if the fact was brought out in court that the defendant belonged to such a union. The lawyers whose opinions were obtained and read by the doctor say it cannot legally affect the case. There is no doubt but that this is true. But the verdict of a jury in malpractice suits is not determined by the legal aspect of the case; and circumstances which cannot have any legal effect upon the case have often made a deep impression upon a jury and decided the case against the physician. To illustrate: In Dr. Bettman's first trial the experts of the prosecution testified so unreservedly in the doctor's favor that had the case been then submitted to the jury without arguments, the doctor would have been acquitted at once. To fortify his cause Dr. Bettman's lawyer called a number of experts whose testimony was of course only cumulative. Now, what did the prosecuting lawyer do? Did he make an effort to break down the expert evidence by scientific arguments? No, sir; but he wiped out its effect upon the jury by the mere waving of his hand, speaking thus: "The defense has piled up a mountain of expert evidence. But, gentlemen of the jury, what does it all amount to? These doctors are working together in the same hospital. Don't you see that they have a common interest to sustain each other, because every one of them may be in the same fix some day? Don't you know they are clannish? They won't admit that one of them can make a mistake. O, no!" One could fairly see the impression this harangue made upon the jury, and they rendered a verdict against the doctor, though it is certain the lawyers will say the fact of his being associated with the experts in the same hospital should and could legally not prejudice the jury. But it evidently did, all the same. And after such experience, can you for one moment believe it would not damage the physician's cause if it was shown he and his experts belonged to a society formed for the express purpose of mutual assistance in malpractice suits. A mighty poor lawyer indeed he would be who could not make a great deal out of it before a jury.

Very interesting was that part of the paper in which the doctor evolved his idea how this new society could prevent, ward off, malpractice suits. He believes the shysters would not be so eager to engage in this business if they knew they had to fight a corporation with plenty of means to employ the best legal talent. Why this should discourage those fellows, it is hard to understand. They do not sue poverty-stricken doctors. Whom they select for their victims they suppose to be rich and consequently able to employ a good lawyer. They do not expect to have an easy game; but why should they not try it? They don't risk anything by it. The blackmailer's stake is only two dollars and a half for filing his application, and his lawyer's stake is his time, which is not worth much anyhow. So, you see, they have nothing to lose, but much to gain. What difference should it make to them whether the opposing counsel is engaged by one physician or one hundred? If you wish to devise means by which this blackmailing nuisance can be stopped, or at least reduced to a minimum, you must try to get at the roots of the evil; that is, you must find the causes which usually bring it forth. And you will not go far to find them; for you find them right at your door, in our own profession, in the shape of *indiscriminate dispensation of gratuitous services and of unkind remarks*

*of one physician about another.* Physicians are altogether too quick to give their services gratis to almost anybody at any time. But you know very well people do not value very much what they can get for the mere asking; they do not think much of what they get for nothing. And it is also a widespread notion (especially among the lower, uneducated people) that the quality of service is regulated by the amount of money they pay for it; that the treatment at a free Dispensary, because gratuitous, is not the same, not as good as at the physician's office where they have to pay for it. These people cannot persuade themselves that a physician will take the same interest in a case whether or not he is paid for his services. The poor, therefore, are always suspicious that they do not get their full share of attention; they are quickly ready to charge their physician with carelessness if the case goes wrong. And with a patient in this frame of mind it takes but very little encouragement to begin a suit for damages. And in nine out ot ten cases, doubtless, this encouragement is furnished by the members of our own profession. He did not mean to charge physicians with purposely, wilfully instigating a lawsuit against a professional brother. Though this has been done, such extraordinary baseness is a rare exception.

What DR. HOTZ had reference to is the inconsiderate, careless, thoughtless habit of expressing an opinion about a case, or a colleague. To illustrate: A physician at a dispensary shows a bad case to professional friends, and, without thinking of the possible evil consequences, makes in the presence of the patient some remark like this: "Well, perhaps I ought to have done this or that." The patient, already laboring under the impression that he was not fairly treated because he could not pay, sees in the doctor's remark the strongest confirmation of his suspicion, goes to a shyster and begins a suit for damages. And, doubtless, in a similar way the mind of a patient is often poisoned and set against his physician by a careless or unkind remark of another physician. So many physicians are always ready to express their opinion about their colleagues in the presence of anybody, or to criticize their professional acts upon the information received from a patient or some old woman. Now you all know how these people misconstrue the words of a doctor; how they pervert the facts inadvertently. You must admit you cannot rely on what patients tell you, and you cannot form an opinion that is worth anything of a case you have not seen or been informed about by the attending physician. Why, then, don't you say so when some busybody asks you what you think about that case of Dr. H.? Or if you know the physician, say he is competent to attend to his own business; if you don't know him, change the subject. But at all events, unless he be a notorious quack, refrain from uttering any words which even only insinuate the possibility of a mistake or want of skill of your colleague.

Stop running each other down; stand by each other; sustain each other, "stick together and be clannish;" let it be understood in public that no reputable physician will prostitute himself by going to court as expert for a blackmailer. If all the reputable physicians of this city adopt and act on this principle, blackmailing the medical profession would soon be a thing of the past, and malpractice suits more effectually prevented than by the organization of a protective union.

PROFESSOR P. S. HAYES said that from his costly experience in a malpractice suit he felt that an association such as suggested by DR. DOERING would be of great service. The lawyer employed by such an association would speedily acquire such a fund of medical knowledge that he would be considered an expert in malpractice cases. He would not require an amount of coaching necessary to prepare for any given case, as would be requisite in the case of a lawyer who had had no experience in such cases. His opportunity for obtaining information in a given case would be largely extended, for each member of the association to whom he might apply would be interested in giving him the desired knowledge. He would soon become acquainted with medical witnesses and know which would give the best testimony in any case.

An association of the character suggested by the paper might be a means of educating its members in regard to laws bearing on the rights of physicians and their patients, now not generally understood.

For one he is heartily in favor of such an association, and should give it his hearty support.

PROFESSOR G. C. PAOLI said DR. DOERING'S paper is not only a valuable one, but contains such a high, noble, charitable feeling, that the Society ought to be grateful to him. He wondered that such steps had not been taken before, because so many of our professional brethren have not only suffered annoyance, but pecuniary loss as well. How can we expect from an ignorant jury a decision based on scientific knowledge and justice?

DR. F. M. WELLER said that the subject of the paper was worthy of consideration; that the discusion of the formation of an association with an object so widely different from the Medical Society seemed out of place; the one essentially scientific, the other in the nature of an insurance. The right to form such an organization was unquestioned; the policy should be considered by each individual. That while any one might be made the object of blackmail, he believed that charges of malpractice more frequently arose from the ignorance of physicians of the statutes affecting the practice of medicine, especially those of the criminal code, and of the rulings of the courts in cases.

DR. H. D. VALIN read a paper on

THE ORIGIN AND SIGNIFICANCE OF THE SEXES AND THE DETERMINATION OF SEX IN UTERO.

He said that it is not generally known that intermediate steps exist by which sexual generation evolved from a primitive mode of conjugation of two or more similar organisms. The process of conjugation observed in the slime molds resembles a sexual process of extreme simplicity. But this union seems to take place between undifferentiated individuals, and as no sexual organ or secretion is present, it could not be called sexual, and it is only a step higher than that observed by Ch. Robin in the noctiluca, individuals of which sometimes swallow others of their own species as they would a particle of food.

The evolution of the sexes is epitomized in the three following species of the volvox family: *Pandorina morum*, *Volvox globator*, and *Volvox aurens*. In the first the spermatozoids are not differentiated from the granules of the ovum, in the second they are so differentiated, and in the third the two sexes exist each in a different colony.

In the animal kingdom, some species of mollusks contain an organ (ovo-testis) from which spermatozoids as well as ova are produced. In many animal species both sexes are contained in one individual, as in the case of tape-worm, leeches, earthworms, barnacles, oysters, some star-fishes; and among the vertebrates such is the case in several species of serranus, as well as in other fishes. However, most of the vertebrated animals have the sexual organs distinct in two different individuals, but a condition closely resembling hermaphroditism often occurs by reversion as an anomaly; and should any one disbelieve that some remote progenitor of the whole vertebrate kingdom has been hermaphrodite, there remain the facts "that at a very early embryonic period both sexes possess true male and female glands" (Darwin), and in each mature individual rudiments of some of the sexual organs of the opposite sex are extant.

The condition of hermaphrodite is advantageous enough as long as the struggle for existence is but slightly marked, but in higher life, where the least advantage is of moment hermaphroditism gives rise to separate sexes in the following manner: those hermaphrodites whose spermatozoids and ova mingle with the generative products of other hermaphrodites breed a progeny which is more vigorous than that resulting from a single hermaphrodite, and thus copulation between hermaphrodites became established in the struggle for existence; but this is an observed fact, for Darwin said: "There is reason to believe that with all hermaphrodites two individuals, either occasionally or habitually, concur for the reproduction of their kind."

Of course that set of organs in one individual hermaphrodite which had been used more frequently would the better perform their function, while the opposite set would become less active; and then we would find some hermaphrodites with better developed male organs; and others with better developed female organs; and the progeny resulting from the union of two such more differentiated individuals would be especially favored in their struggle for life and would breed individuals in which one set of organs would be abortive, while the other would be highly developed, as is the case in as high an animal as man. Such a transitory stage as alluded to above is met in mollusks, and in the common oyster; the genital cœca in any given individual are found to be either almost all ovigerous or almost all spermigerous (Huxley).

In the case of hermaphroditic human persons the organs of one sex almost always predominate over those of the other.

The foregoing facts and inferences warrant me in formulating the following hypothesis, which will explain the law of heredity: In hermaphrodites, while the organs of one sex become more highly evolved, at the expense, as it were, of the opposite sex's organs, this latter set, as embryology proves, is slow to disappear, and concentrates itself in the germinal matter. In other words, as the male organs become better developed, the spermatozoids acquire a faculty for developing a female offspring, and vice versa. This hypothesis is based chiefly on the facts that the queen bee (Huxley) contains a male germ in each of her unimpregnated ova, while the male bee transforms, by means of his spermatozoids, the same ova into females.

*Determination of Sex in Utero.*—In case each sex generally reproduces the opposite, as I claim, it is easy to understand why the number of each remains about stationary under ordinary circumstances. The law of Thury, of Switzerland, that "conception following menstruation produces females, and conception preceding menstruation produces males, harmonizes with my hypothesis if we consider that a greater number of spermatozoids and more active ova will reach the ovum in the uterus than would in the Fallopian tubes and in the ovaries, and should any given cause act to reduce one sex, the other sex would thrive the better and would reproduce a majority of the rarer sex, thus restoring the equilibrium, and insuring an equal number of either sex in the progeny.

There being no discussion on this paper, the society then adjourned.

